# Stem Cell Clinical Trials in Spinal Cord Injury: A Brief Review of Studies in the United States

**DOI:** 10.3390/medicines7050027

**Published:** 2020-05-12

**Authors:** Andrew Platt, Brian T. David, Richard G. Fessler

**Affiliations:** 1 Department of Surgery, Section of Neurosurgery, University of Chicago, Chicago, IL 60612, USA; andrewplatt2014@gmail.com; 2 Department of Neurosurgery, Rush University Medical Center, Chicago, IL 60612, USA; Brian_David@rush.edu

**Keywords:** spinal cord injury, stem cell transplant, clinical trial

## Abstract

**Background:** Although many therapeutic approaches have been attempted to treat spinal cord injury, cellular transplantation offers the greatest promise in reconstituting the architecture of the damaged cord. **Methods:** A literature review was conducted to search for clinical trials investigating stem cells as treatment for spinal cord injury in the United States. **Results:** Overall, eight studies met inclusion criteria. Of the included studies, four were identified as being terminated, suspended, or not yet recruiting. Two studies were identified as currently recruiting, including one phase one trial evaluating stereotactic injections of human spinal cord-derived neural stem cells in patients with chronic spinal cord injuries, and one trial of transplantation of autologous bone marrow derived stem cells via paraspinal injections, intravenous injections, and intranasal placement. One study was identified as an active study, a phase one trial of intrathecal injection of 100 million autologous, ex-vivo expanded, adipose-derived mesenchymal stem cells. One trial that was listed as completed is a phase 1/2a, dose escalation study, investigating stereotactic injection of human embryonic stem cell derived oligodendrocyte progenitor cells. **Conclusions:** Although few significant publications have emerged to this point, current trial results are promising.

## 1. Introduction

The global incidence of spinal cord injury (SCI) ranges between 10.4 and 83 per million inhabitants per year [1,2]. Of these injuries approximately half result in complete neurologic injury and approximately 33% result in tetraplegia [1,2]. SCI has a male predilection and affects 3 to 4 times more males than females [1,2,3]. The mean age of patients at time of injury has increased from 29 in the 1970s to closer to 43 now [1,2,4]. This increase likely reflects the increase in mean age of the general population and greater number of spinal cord injuries that are occurring in elderly populations [2]. There is a significant geographic disparity in cases of SCI which may also be related to discrepancies in reporting [2,5]. A recent study found the incidence of SCI in North America to have increased from 43.3 to 51 per million inhabitants per year and to have increased in Europe from 13.9 to 19.4 per million inhabitants per year over the course of 30 years [1]. During this time frame, however, the prevalence of SCI in Europe and North America has remained fairly stable [1]. The most common cause of spinal cord injuries are motor vehicle collisions followed by falls (the leading cause in elderly patients), violent crime, and athletics.

Although many therapeutic approaches have been attempted, cellular transplantation offers the greatest promise in reconstituting the architecture of the damaged cord by providing a permissive substrate, replacing lost cells, enhancing tissue preservation, supporting axonal regeneration, and modulating the inflammatory response. In particular, mesenchymal stem cells (derived from bone marrow, adipose tissue, or blood) have been reported to enhance neuroprotection, immunomodulation, sprouting, and axonal regeneration. Neural stem cells have also demonstrated these capacities, as well as the ability to form synapses between graft and host, and, in the case of cells directed toward a glial fate, remyelinate. This manuscript includes a review of clinical trials investigating stem cell transplantation for treatment for spinal cord injury.

## 2. Methods

A review of literature was conducted using online databases PubMed, the Cochrane Library, and clinicaltrials.gov to search for clinical trials investigating stem cell transplantation for treatment of spinal cord injury. Keywords “spinal cord injury” and “stem cell” were used to identify studies of interest. Additional manual searches through cited references were performed. Only clinical trials were included in further analysis. Cohort studies, case-control studies, non-English publications, editorials, conference abstracts, errata, book chapters, systematic reviews, meta-analyses, case reports, and case series were excluded. Patient expanded access investigational new drug (IND) studies were excluded. Clinical trials conducted outside of the United States were also excluded. For studies identified through clinicaltrials.gov without associated references, further searches through PubMed were conducted using the trial name and the name of referenced investigators. Studies were evaluated for trial status (terminated, active, recruiting, etc.), clinical trial phase, intervention model (method of group assignment), number of subjects, inclusion criteria, method of transplantation, and primary outcome.

## 3. Results

In total, 106 manuscripts were identified. Several trials were excluded for using non-cell-based therapies, such as Sovateltide and Methylprednisolone, for using non-stem cell-based therapies, such as transplantation of harvested Schwann cells, or for including non-human subjects. Two studies represented Individual patient expanded access investigational new drug (IND) studies and were excluded. Several studies were excluded for being conducted outside of the United States. Overall, eight studies met inclusion criteria (Table 1).

### 3.1. Terminated or Suspended Trials

Of the included studies, two were identified as being terminated. This included a phase one trial of intravenous infusion of autologous bone marrow progenitor cells in pediatric patients. The reason for termination was cited as primary investigator relocation. A literature search was conducted which identified several studies regarding transplantation of autologous bone marrow progenitor cells in patients following traumatic brain injury and one study concerning transplantation of autologous bone marrow progenitor cells for treatment of sensorineural hearing loss, however, no studies regarding transplantation for spinal cord injury [6,7,8,9,10,11].

A second study was a phase two trial sponsored by StemCells, Inc, of intramedullary implantation, a route of infusion directly into the spinal cord, of human central nervous system stem cells (HuCNS-SC) in patients with cervical spinal cord injuries of at least 4 months duration. Only patients with American Spinal Injury Association Impairment Scale A and B (AIS A-B) injuries were included. Thirty-one patients were enrolled in two cohorts. The first cohort included six patients enrolled in a dose escalation study, which allows for the determination of a dosage safety window, with patients receiving 15 × 10^6^ to 40 × 10^6^ stem cells during transplantation [12,13]. The second cohort of patients included 11 patients who were randomized to intramedullary injections of 40 × 10^6^ stem cells [12,13]. There were 18 serious adverse events (SAE) in 12 of the patients who underwent injections [12]. Patients were further assessed by the International Standards for Neurological Classification of Spinal Cord Injury (ISNCSCI) upper extremity motor score (UEMS). Compared to controls, patients following transplantation showed greater improvements in UEMS however the difference was not statistically significant. The trial was ultimately terminated secondary to financial considerations. A second trial of patients with thoracic spinal cord injuries outside of the United States was also carried out prior to early termination for financial considerations. HuCNS-SC have also been investigated for treatment of Pelizaeus-Merzbacher disease, an x-linked recessive leukodystrophy that leads to hypomyelination in the central nervous system, and neuronal ceroid lipofuscinosis [14,15,16]. 

One study was identified that was suspended secondary to financial considerations. The study was a phase one trial of intrathecal infusion, a route of infusion into the subarachnoid space, of autologous bone marrow-derived mesenchymal stem cells. A literature search identified studies investigating transplantation of stem cells for treatment of myocardial and limb ischemia, however, no studies regarding transplantation for spinal cord injury [17,18,19].

### 3.2. Trials Currently in Recruitment

Two studies were identified as currently recruiting. One study, sponsored by Neuralstem Inc. (now rebranded as Seneca Biopharma Inc.), is a phase one trial with single group assignment evaluating stereotactic injections of human spinal cord-derived neural stem cells. The trial is evaluating patients with chronic (1 to 2 years post-injury) AIS A spinal cord injuries. As a phase one trial the primary outcome measure is adverse events and clinically significant laboratory abnormalities within the first six months following injection. In total, the study will include eight patients broken into two groups. The first four patients were treated following spinal cord injuries of the thoracic spine (T2–T12). The second four patients will be enrolled following injuries to the lower cervical spine (C5–C7). Prior to beginning the trial, the NSI-566 human neural stem cell line showed favorable results in in vitro and animal models and was approved for a phase one and two trial in the treatment of amyotrophic lateral sclerosis (ALS) [20,21,22,23]. The results of the ALS trial, which included dose escalation, showed spinal cord transplantation of human stem cells could be carried out in a safe manner at escalating doses in both the lumbar and cervical spine. Results from the first four patients, all thoracic, following transplantation for SCI are promising. No serious adverse events were reported in the first 18–27 months post-procedure, and two patients showed one to two levels of neurologic improvement [24]

Another study that is currently in recruitment, is a trial of transplantation of autologous bone marrow derived stem cells via bilateral paraspinal injections of cells at the level of the injury as well as superior and inferior to that spinal segment. Patients will also receive an intravenous injection and intranasal placement of stem cells. The trial has a parallel assignment model and will assign 40 patients to three treatment arms: treatment only, treatment + exoskeleton movement, and treatment + virtual reality visualization. A literature search identified studies investigating transplantation of stem cells for treatment of several ophthalmologic conditions, however, no studies regarding transplantation for spinal cord injury [25,26,27,28,29,30,31,32,33].

### 3.3. Active Studies

One active study that was identified is the CELLTOP trial, a phase one trial of intrathecal injection of 100 million autologous, ex-vivo expanded, adipose-derived mesenchymal stem cells. The trial is evaluating patients from two weeks to one year post-injury who are AIS A or AIS B for incidence of acute adverse events up to four weeks following transplantation. Previous in vitro and animal model studies have shown exceptionally promising results following transplantation of adipose derived mesenchymal stem cells [34,35,36,37,38,39,40] A recent publication from the CELLTOP trial examined the first treated patient. The patient was initially an AIS A spinal cord injury following a surfing accident, however, improved to AIS C prior to enrollment in the trial. Following implantation, the patient did not suffer a safety issue or adverse event during 18 months of follow-up. The patient was also noted to show improvement in motor and sensory scores during the follow-up period [41]

### 3.4. Trials in Pre-Recruitment

One trial is listed as not yet recruiting. It is a phase two trial assessing injection of umbilical cord blood mononuclear stem cells into bilateral dorsal root entry zones above and below an area of chronic (>1 year) spinal cord injury. Twenty-seven patients will be randomized through parallel assignment to three groups. Both groups A and B will receive stem cell transplantation. Group A will further receive lithium carbonate (750 mg/day) whereas group B will receive a placebo. Lithium has been shown in vitro to stimulate stem cell proliferation, neurogenesis, and regeneration of long spinal tracts, and has been shown in an animal model to improve locomotor recovery after transplantation of umbilical cord blood mononuclear stem cells [42,43]. Group C is a control group. The trial was double blinded except for group C.

The trial followed phase one and two clinical trials which were conducted outside the United States. In the phase two trial, 20 patients with complete chronic spinal cord injuries were assigned to five treatment groups. The first three groups received escalating dosages of umbilical cord blood mononuclear stem cells. Group D received the dosage of group C with addition of a bolus of methylprednisolone (30 mg/kg). Group E received the dosage of group C with the addition of a bolus of methylprednisolone and a 6 week course of lithium carbonate. During the follow-up period, 68 adverse events were recorded, all resolved with routine therapies. Three patients in the phase two trial had severe adverse events including slow wound healing, cerebrospinal fluid leak and wound dehiscence, and deep venous thrombosis. AIS scores improved from A to B in two patients and from A to C in three patients. In comparison to baseline, patients showed statistically significant improvements in walking index of spinal cord injury scores and spinal cord independence measure scores as well as in bladder and bowel function. Differences among treatment groups were not significantly different [43]

### 3.5. Completed Trials

One trial that was listed as completed is a phase 1/2a, dose escalation study, investigating stereotactic injection of human embryonic stem cell derived oligodendrocyte progenitor cells. The study evaluated patients with AIS A or B cervical spinal cord injuries who were 21–42 days post-injury. Patients were assigned to one injection of 2 million or 10 million cells, or 2 injections of 10 million cells for a total of 20 million cells. The primary outcome assessed was the number of adverse events that occurred within one year of injection. There have been no significant adverse events to date, however, full results from the trial have not yet been published. Animal testing prior to clinical trials showed significant improvements in remyelination and recovery of motor function after transplantation of human embryonic stem cell derived oligodendrocyte progenitor cells at 7 days following spinal cord injury. These results were, however, not shown at 10 months post injury [44,45,46,47]. Although not yet published in the peer reviewed literature, public announcement by the sponsoring industry reported significant improvement in two-thirds of the patients. Results from an earlier phase 1 trial, in which five patients with AIS A thoracic injuries underwent stereotactic transplantation between 7 and 14 days post-injury were positive. There were no surgical complications and no severe adverse events.

## 4. Discussion

A literature search was done to identify clinical trials conducted in the United States investigating stem cell transplantation for treatment of spinal cord injury. Laboratory testing and animal models were used to test each stem cell type prior to human clinical trials. This study included only trials registered in the United States with approval from the United States Food and Drug Administration (FDA). This was done in-order to investigate trials that had all gone through the same approval process, thus making results more standardized and directly comparable. Regulatory requirements outside the United States vary by region and are generally less stringent than FDA requirements [48]. In comparison to approval in the European Union, the approval process in the United States is also faster and more transparent. Whereas all non-published data in U.S. trials is available for review online and by request, in the European Union, non-published data is considered “commercially sensitive” [49].

Of the eight studies that met inclusion criteria two studies were terminated and one was suspended. Five studies remain that are currently in pre-recruitment, recruitment, active, or completed. Of the five studies, two included transplantation of mesenchymal stem cells including autologous, adipose derived cells, and autologous bone marrow derived cells. One study each included transplantation of neural stem cells, umbilical cord blood cells, and embryonic stem cell derived oligodendrocyte progenitor cells, respectively.

Several factors varied across studies including the method of cellular transplantation. The majority of included trials include intrathecal or intramedullary transplantation with intravenous administration being less common. Intravenous transplantation has the benefit of being the least invasive although also has the significant disadvantage of not directly implanting cells within the subarachnoid space. Intramedullary transplantation is the most invasive, has the greatest surgical risk, however, has the greatest potential for long-term engraftment [12]. It can be carried out in a free hand technique or through stereotactic implantation. There is no clear superiority between different transplantation methods at this time as few studies have directly compared them in human studies.

A majority of US trials in this literature search have shown promising results. The StemCells, Inc. trial showed feasibility of freehand intramedullary injection of human central nervous system stem cells. Following a successful dose escalation study, patients showed greater improvement in upper extremity motor score in the transplantation group prior to study termination. Results from the NeuralStem and CELLTOP trials are also promising although both trials have not shown full phase one data. Positive clinical results from the dose escalation study of AST-OPC1 are likely to lead to the first phase three trial in the United States of stem cell transplantation for SCI. 

## Figures and Tables

**Table 1:** Summary of included studies

Identifier	Trial Name	Status	Intervention	Phase	Intervention Model	N	Inclusion Criteria: Age (Years)	Inclusion Criteria: AIS Scale	Inclusion Criteria: Time from Injury	Transplantation	Primary Outcome
NCT01328860	Autologous Stem Cells for Spinal Cord Injury (SCI) in Children	Terminated	Autologous Bone Marrow Progenitor cells	1	Single Group Assignment	10	1–15	A–D	6 months to 4 years	Intravenous	AIS
NCT01162915	Transfer of Bone Marrow Derived Stem Cells for the Treatment of Spinal Cord Injury	Suspended	Autologous bone marrow-derived mesenchymal stem cells.	1	Single Group Assignment	10	18–65	A	2 weeks to 60 months	Intrathecal	Safety
NCT03308565	Adipose Stem Cells for Traumatic Spinal Cord Injury (CELLTOP)	Active, not recruiting	Autologous, Adipose derived Mesenchymal Stem Cells	1	Single Group Assignment	10	>18	A–B	2 weeks to 1 year	Intrathecal	Incidence of acute adverse events
NCT01772810	Safety Study of Human Spinal Cord-derived Neural Stem Cell Transplantation for the Treatment of Chronic SCI	Recruiting	Human spinal cord-derived neural stem cell	1	Single Group Assignment	8	18–65	A	1 year to 2 years	Intramedullary	Adverse events and clinically significant laboratory abnormalities
NCT03225625	Stem Cell Spinal Cord Injury Exoskeleton and Virtual Reality Treatment Study (SciExVR)	Recruiting	Autologous bone marrow derived stem cells	NA	Parallel Assignment	40	>18	A–D	NR	Paraspinal, Intravenous, Intranasal	AIS
NCT02163876	Study of Human Central Nervous System (CNS) Stem Cell Transplantation in Cervical Spinal Cord Injury	Terminated	Human central nervous system stem cell	2	Randomized Parallel Assignment	31	18–60	B–C	>12 weeks	Intramedullary	ISNCSCI upper extremity motor scores
NCT03979742	Umbilical Cord Blood Cell Transplant into Injured Spinal Cord with Lithium Carbonate or Placebo Followed by Locomotor Training	Not yet recruiting	Umbilical cord blood mononuclear stem cells	2	Randomized Parallel Assignment	27	18–60	A	>12 months	Intramedullary	Walking Index of Spinal Cord Injury (WISCI II)
NCT02302157	Dose Escalation Study of AST-OPC1 in Spinal Cord Injury	Completed	Human embryonic stem cell derived oligodendrocyte progenitor cells	1/2a	Single Group Assignment	25	18–69	A–B	21–42 days	Intramedullary	Adverse events
